# Bereaved Parent Perspectives on End-of-Life Conversations in Pediatric Oncology

**DOI:** 10.3390/children9020274

**Published:** 2022-02-17

**Authors:** Rhonda Robert, Shehla Razvi, Lisa L. Triche, Eduardo Bruera, Karen M. Moody

**Affiliations:** 1 Division of Pediatrics, The University of Texas M.D. Anderson Cancer Center, 1515 Holcombe Boulevard, Unit 87, Houston, TX 77030, USA; srazvi@mdanderson.org (S.R.); lltriche@mdanderson.org (L.L.T.); kmoody@mdanderson.org (K.M.M.); 2 Department of Palliative, Rehabilitation and Integrative Medicine, The University of Texas M.D. Anderson Cancer Center, Houston, TX 77030, USA; ebruera@mdanderson.org

**Keywords:** caregiver experience, child, cancer, end of life, palliative care, patient experience, pediatrics

## Abstract

Background: Professional education pertaining to end-of-life care with pediatric oncology patients is limited. Pediatric trainees learn about end-of-life conversations largely from the provider’s perspective. Bereaved parents can inform the education of oncologists and the interdisciplinary team by sharing their perceptions and preferences through personal narratives. Methods: The aim of this project was to enhance the healthcare teams’ understanding of bereaved parents’ end-of-life care preferences through narratives. Bereaved parents were recruited from our institution’s Pediatric Supportive Care Committee membership. Parents were tasked with identifying elements of care that were of the greatest importance to them, based upon their personal experiences during their child’s end-of-life care. Narratives were analyzed using standard qualitative methods. Results: Parents of five patients participated, including four mothers and three fathers. Ten themes summarizing essential elements of end-of-life care were identified, including early ongoing and stepwise prognostic disclosure, honoring the child’s voice, support of hope and realism, anticipatory guidance on dying, and continued contact with the bereaved. Conclusion: Bereaved parents emphasize the need for providers to have ongoing honest conversations that support realism and hope that can help them to best prepare for their child’s end of life and to remain in contact with them after death.

## 1. Introduction

Palliative care is an integral part of pediatric oncology practice and pediatric hematology/oncology fellowship training. However, pediatric oncologists receive minimal to no formal education on end-of-life conversations [1]. Training materials and conversation guides are more readily available for initial conversations pertaining to cancer diagnosis, prognosis, and treatment planning than for end-of-life conversations in this context. Much of what pediatric trainees learn about end-of-life conversations is provided through role modeling or written information, typically from the provider’s perspective; very limited information has been derived from patients’ and caregivers’ perceptions and preferences and translated into practice [2]. Patients and family members are rarely included in the development of training guides, yet they may serve as important teachers [3]. Narrative-based medicine is an approach that can be used to gain a broader and deeper perspective of the patient experience [4].

Families plan according to end-of-life conversations, including those with professional caregivers. In order to enhance clinical provider end-of-life communication, bereaved parents serving on our Institution’s Supportive Care Council volunteered to present their narratives using a grand rounds approach on their experiences with and insights into end-of-life communication in pediatric oncology. Herein, we report our analysis of these informative narratives.

## 2. Methods

All of the participants were parent members of our institution’s Pediatric Supportive Care Committee (PSCC), a family-centered care committee consisting of both parents and oncology professionals. Membership was balanced equally between parents and oncology professionals, with a target group size of 6–8 in each category. Professional disciplines represented on the PSCC included physicians, registered nurses, social workers, advanced practical nurses, physician assistants, psychologists, child life specialists, and chaplains. Parent members each had a child who had been treated at the institution and died from cancer at least 1 year prior to the parent joining the committee (see Table 1), lived in the greater metropolitan area where the hospital is located, committed to a 2 year committee service term, and attended monthly meetings.

The committee had formed over 10 years earlier and was aimed at improving end-of-life care for patients and their families. The parent members established an annual agenda, which included educating clinicians during the course of the institution’s Pediatric Grand Rounds. This was a first such event for our program. Communication, particularly end-of-life conversations, was a priority training topic for the committee. For this Pediatric Grand Rounds, the format was a 1 h parent panel moderated by two oncology professionals. Parents were tasked with identifying elements of care that were of the greatest importance to them, based upon their personal experiences during their child’s end-of-life care. All resulting narratives and dialogue between PSCC members were recorded and transcribed.

All parent members of the PSCC were invited to participate, and these parents were representative of the PSCC. Parents of five patients participated, including four mothers and three fathers. These parents willingly offered this tremendous learning opportunity, hoping that professional caregivers would learn from their perspectives and develop strategies for effectively dealing with future families. One parent expressed his inspiration to participate as a “way of passing on some of our experiences in hopes of helping you to make the hospital the best place in the world”.

The University of Texas M.D. Anderson Cancer Center Institutional Review Board (IRB) assigned this report a determination of not human research; therefore, IRB approval and ethical review were waived. Each participant provided authorization to release deidentified information. The block quotations are from the parent panelists.

## 3. Data Analysis

The transcripts were evaluated using common summative techniques for coding qualitative data, namely, open coding followed by axial coding [5]. Specifically, the narratives were reviewed (first by R.R.) line-by-line and broken down into smaller chunks of information representing a single idea and given a label that described the idea. This was repeated until all of the transcripts were reviewed, sorted into chunks, and labeled. Then, the labels were reviewed along similar and different properties and dimensions and reorganized into groups and relabeled as needed (open coding). This relabeling allowed for splitting and/or lumping of categories as directed by the data in order for it to fit nicely into a particular category. Data were then reanalyzed within the categorical structure to develop a contextual framework for the categories (axial coding). The data, categories, and contextual framework allowed for the emergence of themes. A second analysis undertaken by S.R. independently proceeded with the same analysis procedure, before she presented her findings to the primary investigator (R.R.). Subtle differences in the concept dimensions were handled by consensus.

## 4. Results

Ten themes emerged from the analyses. The resulting themes are summarized in Table 2. The themes were grouped according to their temporal relevance to death. Narratives aptly illustrating the themes that emerged from the qualitative analysis are included.

*Theme #1. Invite open, ongoing communication with checking for understanding; this is not a one-time conversation*

Bereaved parents expressed a need for ongoing, open communication with assessment of understanding about their child’s prognosis. This type of communication allows parents time to absorb the information at their own pace and for the medical team to have insight into the parents’ process. They acknowledged the difficulty for staff to have these conversations and the strong emotions that come up from parents and encouraged providers to persevere.

“This is not a one-time conversation. It’s not just a talk that you are going to have. This is a dialogue that needs to come from your team. I know for us, my wife and I heard things very differently at different times. A lot of times, I didn’t hear what was being said very well. I wasn’t always good at that for whatever reason. I knew exactly where things were headed. In the moment, I didn’t want to hear it. I didn’t want to accept that this was the path that we were going down. So, parents are going to react like that; one parent will hear one thing and the other parent will hear another thing. The child might hear another. So, you’ve got to be aware of that and repeat yourself”.

“I just want people to be more comfortable talking about death. Honestly, it’s just a profound gift that you can give to someone to make their end of life as beautiful as the beginning. Really think of it as that opportunity that you can just make such a difference. I will never ever forget the people who were a part of our lives during the time that our son died. Ever”.

“Don’t be afraid of us. We might yell and scream at you. But it really has nothing to do with you; it has to do with the fact that our child is dying. Let us own that and let us hear what we need to hear so that we can move forward”.

“Say the word ‘cancer’ to us, and our minds immediately say, ‘Our child could die.’ We say that in our head, whether we say it aloud or not. But you have to be willing to let us have that kind of conversation. I know that’s uncomfortable for some of you. Some of you do that better than others, I’m guessing. But you don’t have a choice because we have to face that. So, if at any point you can say to us, ‘Yes, this does happen, but here’s what we’re going to do’; or, “Here’s what we can do to try and make this better”; or, “Here’s what we can do to make life as good as it can be, for as long as it can be”; or, “Here’s what we can do to help you to help your child own what they can”.

“Things were going pretty well, but then suddenly she took a turn for the worse. When we were in ICU and the physician on call that night was called to talk to us about 4 in the morning to discuss DNR, regarding resuscitation plans, that’s when the DNR conversation occurred, at 4 o’clock in the morning, with no conversation leading up to that. That was the very first conversation that any staff person had had with us. I’m an attorney; I do guardianship work; I know all about DNRs. But no one had ever talked to us about it in the context of our child. And while I have to say that the physician did an excellent job, especially having been awakened at that hour in the morning and having not been the treating physician and not really knowing us, it would have been better, I’m convinced, if there had been a series of conversations. I think that this is a very difficult piece of information to convey to the family… that your child is not going to make it and you need to make this decision about whether to use life support or not. And so, ideally, I think it would be best if there were a series of conversations”.

“We figured out about a month before our son died, there were no more treatment options. We had about 6 weeks where he got to do whatever he wanted. And we all got to hang out. So, to delay a conversation with a family is the worst thing you can do. The sooner you can give that family the opportunity to have that conversation, the better off the patient is and the better off the family is. In the course of this, I have met lots of families who have unfortunately lost their children. There’s one distinct thing that separates families. It is if a child has died suddenly versus having a little bit of time to prepare. There’s a big difference in how a family deals with it, when they aren’t given a little bit of time to prepare. So, if you can give that to a family, it makes a big difference”.

*Theme #2. Enable stepwise disclosure; allow parents time to process bad news before telling the child, and allow the child to have a voice once told*

“I would say too, for our experience, typically when the doctor wants to deliver really bad news to you, you have to have an appointment. So, just a little tidbit, sometimes it’s better not to have an appointment. Sometimes it’s OK to have a chat on the phone to hear, ‘It’s not looking good, the results aren’t good.’ You kind of have to have that in mind before you go to the appointment. It allows you to prep your child. So as much as we have an opportunity to prep our children, the better”.

“I needed to know from doctors what could happen and the options [decisions] I could face, as they would come up—whether good, bad, or indifferent. By the time we finally got to the end, my son had asked, ‘Please shoot me with all the chemo you got and let it try and work.’ He was told, ‘I’m sorry, there is nothing we can do.’ I hit the floor. I did not know what to tell him. I didn’t know what to do. I had no clue there was nothing else to do. He knew he was going to die. We didn’t know the exact time frame but neither did anybody else. But to say to him there was nothing else they could do—I didn’t know how to help him. My feeling is that if there is devastating news, give the parents a heads-up, even when the child wants to know. My son wanted to know everything that was going on. But I couldn’t help him because I couldn’t help myself. I didn’t have enough time. I didn’t know what to say to myself. So, I want the people to know that when things are not looking the way that you expect or hoping or whatever, give everybody enough time somehow to let the family cope. I don’t care if it’s just a little while, to get a grip on it. The kids are having enough problems and you’re having enough problems. Informing them and you [child and parent] at the same time is extremely hard”.

“You know, the hardest thing for our kids is, I’m assuming this for small children as well, but especially in teenagers, it’s the total lack of control. Everybody makes decisions for them, and you can help us by allowing our children to have some choices”.

“Our son died when he was 13 years old. It was pretty apparent that gliosarcoma was not a survivable illness. And so, one of the things that we were able to do is when our son was feeling good, we made some decisions with him about how he wanted to end his life. He personally had had a friend that was kept on life support for almost 3 months. And he said, ‘Whatever happens, that’s not what I want to have happen.’ He was very clear about that, and most of us don’t like to talk about those things even as grown-ups. But we all want to have some degree of control about how we end this life. So, I’m very grateful to the doctors who we talked through it, we wrote it down, and our family was aware of it, and it made a big difference. So, at the end, he was a little less afraid”.

“We were fortunate, our daughter’s physician said to me from the very beginning that we were moving forward, and we’re going to do everything that we can. But if our daughter, who at that point was 13, said, ‘I don’t want to do this anymore,’ and not just because she was mad [you know they do that all the time], but if our daughter seriously said that she didn’t want to do this anymore, I would have a very hard time going against that. I said, ‘So would I.’ But she laid that out for us from the very beginning. Then everything we did moving forward, we had everything on the table”.

“For our experience, we made a decision to have our son make his plans. We knew what his plans were. We moved forward with a signed DNR that we kept with us. It came down to every single treatment was going to make him significantly worse, it was not going to do anything to improve the quality of life, and there was no cure. So, our experience when we went home, our son wanted to die at home, we chose to leverage hospice”.

“So, when our daughter would ask the question, ‘Am I going to die?’, which she would ask from time to time, my answer usually would be, ‘Not today, and we’ll figure out what that means.’ When she chose to stop treatment, she knew that she was going home to die. We had that conversation too. She knew that she was supported here and at home. She knew she was cared for and left this world far more peaceful than the whole time she was in treatment”

“But at the end, he got his wish. He definitely ended his life the way he chose and shut down the way he wanted to”.

*Theme #3. Ensure the availability and alignment of a multidisciplinary team*

Parents reported on the need for more psychosocial support, especially as end of life approaches, and for members of the team to give consistent information.

“You need to plan, think through it, and figure out who your team is and how they are going to best put together a plan for that family”.

“I am very pleased that the pediatric palliative care physician is now having regular appointments in the clinic, so that families might discuss end-of-life issues. I think it’s important for other treating providers, perhaps a psychologist, social worker, chaplain, to be involved. There needs to be a team approach to talk to these families about what is likely to happen, and how they can best handle this horrible news”.

“Different families want different things… I think what distinguishes hospitals and individuals is their ability to actually find out and share what families are actually looking for and ways to make it happen. That can be done on an individual basis but also through the hospital being better organized to make those things happen”.

“If you guys could also be on the same page, that’s real helpful”.

“We had a friend whose son passed away here in ICU, maybe a year after our daughter, I guess. He was ICU, his brain was shutting down, and his mom knew it. She was ready to turn off the machines because she had talked to a couple of the doctors who had said that he was not going to recover the brain functions. Then, another doctor came in and said, ‘If we let him rest for a while, it could improve.’ So then what is she going to do? When one doctor comes in and says ‘yes’ and all the others have said ‘no,’ you can’t ignore that! As a parent, how do you shut off the machine if you have somebody saying that he might get better? So, if you all could talk to each other that would be real helpful. Our doctors did that, and we were really fortunate”.

*Theme #4. Discussion of all options for treatment, including second opinions; be willing to try new treatments*

“Our daughter knew she was a guinea pig in the beginning, and we used to talk about that because everything was ‘they don’t know but try it anyway’”.

“We knew from the very beginning the chances of our daughter’s survival were pretty nonexistent because of how old she was for the disease. But you all were willing to give it a shot and try some things that hadn’t been tried”.

“One of the things that we appreciated the most was that we had gotten second opinions at other hospitals, about a year before our son died”.

“My son passed away here. He had leukemia. Exactly 1 year, almost to the day, the cancer came back. I am very thankful that we had a chance at a second transplant. We knew from the beginning our chances were very slim of him surviving. And of all things, I am so thankful for the time we did have, and the things we got to do and to try”.

*Theme# 5. Support hope, as well as realism; hope can be for different things (when cure is unrealistic)*

“Hope was always something that was on the table, but reality was there as well. Hope and reality have to go side by side. We were fortunate to have that the whole time. I have spent time with families who unfortunately didn’t have that. They didn’t have doctors willing to just be really honest about the reality of the situation and yet remain hopeful. They didn’t know what to do with that and so for me, what I can tell you is that to give us hope isn’t to say that ‘We can fix your child’”.

“Because we knew, and we always had hope, and we knew that the hope was not a cure. So, if you can give us hope for the little life that we have, then great. But don’t give us false hope”.

“And so for us, the hope was that we would have a few more days that she would feel good. That we could go and do things with her. That she could go to school and be a kid—that was our aim. Our daughter got to go to school for day 1. She’d go to school, then come here a few weeks, and then go back to school for 3 weeks. We were fortunate. We were never left without hope, but we were never left believing something that wouldn’t happen”.

“Wants and wishes change as treatment goes on. In the case of our family, as it became clear that our daughter wasn’t going to recover, our hope and wish as a family was that she would be able to come home and spend family time with us, without all of the doctors around”.

*Theme #6 Support for conversation on goals of care and advance directives*

Just as there was always hope for something (even if not for cure), parents reported that, when given honest prognostic information, they could prioritize and plan for goals related to quality of life instead of cure.

“He graduated from elementary school; he had his fourth of his six brain surgeries the very next day. We barely got him through his graduation ceremony. He was determined that he was going to walk with his buddies. We really appreciated it. One of the doctors said, ‘Well, he has to go to his graduation. We’ll just we move his surgery.’ And they did. Those are the kinds of decisions that helped make his life better and for that we are grateful”.

“Even then, we knew she would not survive her disease, but we are going to do what we can to make her life as good as it can be, for as long as we can. That’s when we moved forward”.

“In our case, we asked for a DNR. We were on the second transplant, and we knew our chances were not great. I was kind of told not to worry about it. I’m sorry, I was very worried about it. That was my deepest, darkest fear, to have my child hooked up to all these machines when he’s always talked about not wanting that. I made sure that we had all paperwork on hand, as far as the DNR”.

“Control may be a little bit too strong of a word in the circumstance, but certainly empowerment. The reality of hospitals is that you are told what you have to do and when you have to do it. That’s pretty messed up in the final times. In the final times, families, patients in particular, need to have some empowerment. Empowerment is very important”.

“We went home; she wasn’t expected to live more than a week or two without any potassium; rumor has it we can’t live without potassium. She lived four and a half weeks because she’s stubborn and planned her own funeral. She talked to us the day before she was gone. She talked to us on Father’s Day. She died at 4 in the morning the day after that, on Tuesday morning. We were very blessed to have her as long as we did. She was cared for very well; she went out the way she chose to go out, and we could not ask for more”.

“He wanted to die at home. I wanted that paperwork so that, if we made it home after the transplant, we could do it. Every parent and family is different. You have to go with what the families think. I pre-thought everything out. I’m such a stickler about everything being organized. I have to make sure that I know what’s going on”.

*Theme #7. Provide anticipatory guidance on the process of dying, from physical changes to facilitating closure between the dying child and their siblings*

“The other thing I would say that could be really helpful is to just help us understand death a little better. I know this is really morbid, but I’ve never seen anyone die. I’ve never been a part of that before. If you’ve never personally experienced a patient die, I encourage you to talk to someone who has about what happens. What happens to the physical human body. Hospice gives you a little booklet that explains that the breathing is going to change. But, OH NO, that did not articulate what was going to happen. You can really do a favor to the family, once they are ready, to explain what that might look like”.

“Toward the end of life, there are certain changes that occur in your feet and legs. They turn blue and purple. That is something that you’ve got to tell families. Because that’s a really big clue that it’s getting very close. We don’t know when it’s getting close. When our son’s breathing changed for the first time, we didn’t really get it. We thought, this is it. No, not yet. You really need to help people understand what physically happens at the end of life. As you talk in your teams, I think it’s beneficial that you share how you are going to help the family understand how the physical changes might occur. Not every family will want to hear that. But at least have a conversation among your team for what might happen. We had multiple conversations, in person, over the phone, and that paved the way nicely. It helped our son. Think, at the end of all of this, you have a patient who is really scared to die. I’ve never died before; I can’t really help out. I have no frame of reference. All I could do was what my faith told me might happen or what someone shared with me”.

“Our children really look to us to say, ‘So, what’s going to happen is …’ because that’s what we have always been there to tell them. Think about what you as a team might come up with and what those plans might look like and the little, small things, like telling the siblings, taking off the gowns, really talking about what’s going to happen, and what’s going to happen makes such a huge difference in the lives of the people that you are touching”.

“One thing that happened is that the psychologist at that time came to us. We were all huddled in ICU and said that she thought it would be good for them to talk to our older daughter, who was 12 at the time, and try to help prepare her for what was about to happen. The psychologist and perhaps a child life specialist, I’m not sure who else was involved in that conversation with our other daughter, but they had a conversation with her. And a decision was made, and I’m sure it was our daughter’s decision, that she wanted an opportunity to say goodbye to her baby sister. They came back to the hospital room and asked if we would be willing to leave so that our daughters could have some time alone with one another. I’m so grateful for that. It was not anything that I would have thought of on my own under those circumstances, but I think that it really meant a lot to our older daughter. It’s not just the parents you need to think about, it’s the siblings as well. How do you let them know what’s about to happen? And help them through this most difficult time in their whole life?”

*Theme #8. Comfort the dying child regardless of location at end of life*

For some parents, the hospital was the preferred location at end of life, and it was expressed that hospitals should accommodate this preference. However, it was also noted that hospitals may also want to consider ensuring that their policies support quality of life for dying children.

“My wife wanted to make sure that our final time with our daughter was together and private. I’m pleased to say that the hospital was able to make that happen. But I think it was quite difficult for the hospital to make that decision for various practical reasons. So my first point is that it happened because various individuals made it happen. Individuals kind of went beyond what they needed to do”.

“The other thing that I witnessed was that the patient was in pain and the patient expressed the pain, and the nurse was like, ‘You just had a dose.’ It doesn’t matter anymore. You need to do a better job of reminding each other that it doesn’t matter; go get him what he needs. This is a no-brainer”.

“I was here recently visiting a patient in the hospital. It was the day before he died, and it was obvious that he was dying. This is a small thing, but everyone was still wearing gloves, masks, and gowns. Hello? It doesn’t matter anymore. No one could really touch him. You forget at the end that that human touch and connection is a very important and powerful thing. I sat there and told him a story and people were like, he’s not really hearing anything. I told him a story, and he sat up and said, ‘What do you think that means?’ as clear as a bell. They are there and they are listening and want to be loved and touched. So, at least take the masks and the gowns off”.

“When it became clear that our daughter wasn’t going to be cured, we didn’t want to go to a hospice. This was our action. We wanted the hospital because you were part of our journey. So, I would encourage you to make sure that if family wants you, that you stick with them, continue to be part of them, if that’s what they want. Because certainly, in our case, that was a big deal. And I thank everybody for making that happen for us”.

“We were so grateful that the decision was made to take her to the ICU at the hospital because this is a familiar place, a place where we felt loved. We felt that everyone from the president down to the custodian was compassionate, cared, would smile at us, hug us, do whatever they could to help us; and so, it was truly a blessing that we were here for those last couple of days. My mother who was with us in the room says approximately 150 of the hospital staff members came to our daughter’s room in ICU to tell her goodbye and to support us. That was the worst time in our lives, and I truly, truly appreciate that. I am truly grateful to the staff and what we called our extended family at the hospital”.

“One of the things we found is that being the best in the world, means not just being the best in the world at curing cancer, but being the best in the world at all stages of cancer care, right through the end of life”.

*Theme #9. Support bereaved parent and provide opportunities for them to support other parents*

“The other thing I would say is, and I wouldn’t necessarily have anticipated this but, on the day I walked out of the hospital, on the day that our daughter died, it was a very surreal experience. You see people walking around, moving about, going to their cars, and you just can’t understand it. Because to you, the world is empty, or it seems to have ended. One of the things that kept coming to mind is that not only is our daughter gone but my family at the hospital is gone too. It felt like, you know, who am I? Where am I going? I’ve lost my child and my extended family. So, by participating in this community, we are able to keep that connection with the people here at the hospital, and that means so much to me”.

“Since then, you all have been nothing but a support to me and you will continue to be a part of our family”.

“But, also, we definitely have empathy for the families who are going through the processes that we went through. I think it helps us to help them. It helps us with our healing process. It helps us feel like we are doing something to help someone who is walking the same path that we have previously walked”.

“I assemble the packet that is sent out to the parents after the child has died. To be honest, nothing makes me feel happier than to be involved in that. Help make them feel a little bit better and know somebody still cares about them and that their child died. I send them out a card that reminds them that their child was thought of. When I got my card, I thought it was the most wonderful thing. The things in the [bereavement] packet were very, very informative. Right after my son died, I’ll be really honest, I couldn’t open it; I couldn’t look at it; I just couldn’t do it. A couple of months later, I opened it up. It helped with the problem I was having with my son. But I had to sit and do it on my time. So, I keep coming back because I think it’s a wonderful thing to pass on. To make sure that the parents know that they are all loved”.

*Theme #10. Honor their child’s legacy and keep their child’s memory alive*

“Other than the birth of their child, it’s the most memorable experience they’re going to have about their child. Death deserves just as much attention as birth does”.

“The last week that our daughter was alive, she did a lot of talking about things. Dying wasn’t what frightened her the most. What frightened her the most was being forgotten. If you had ever met her, you would know that’s not a possibility. Being forgotten and being meaningless”.

“So, for me to be here and be able to talk to other parents and to you all and be available in any way that we can, for me, that’s a way to introduce you to people like our daughter. You get to know our daughter whether you like it or not. That’s keeping my promise to her, and it’s allowed what we went through and what she went through to have significance beyond her own life. That’s really what mattered to her; it was her greatest fear. I can honor that by making sure that it doesn’t happen”.

“I think I can say that one of the very most important things to parents who have lost a child is to keep that child’s memory alive”.

Beyond the qualitative analysis of the themes, involved providers who had impacted the families near or at the time of their child’s death were recognized by parents. This recognition was inspirational and encouraged the PSCC to develop additional palliative care educational opportunities. Participants requested future grand rounds be provided by the PSCC, which was accomplished.

## 5. Discussion

This report describes the narratives presented by bereaved parents on end-of-life communication during a Pediatric Grand Rounds. These narratives include 10 essential end-of-life care elements, which may serve as teaching points to guide interdisciplinary team members’ professional development at the time of need. Quality end-of-life patient care involves a complex skill set. Some of these skills are not standard components of medical training programs, but rather of psychosocially oriented professions. Despite the growing availability of educational curriculum and literature, end-of-life care skill development is largely self-directed and on-the-job training.

Feraco and colleagues [6] categorized change consequent to training as attitudinal, knowledge, and skills-based. Attitudinal change or “buy in” oftentimes precedes skill acquisition. A related rhetorical question might be, “Why learn how to facilitate an end-of-life conversation unless the need for the skill is identified and valued?” Indeed, one must identify and recognize the value of the skill of facilitating an end-of-life session before seeking to learn how.

Enlisting the aid of bereaved parents to foster the education of healthcare professionals in end-of life care is a relatively recent and useful development in pediatric oncology. Due in part to this work, the parent advisor voice has been increasingly present in our institution’s educational opportunities. Bereaved parents may in fact be the best messengers to relay the importance of getting this communication right [7].

Some of the themes presented by our parent panel have been noted by other studies of parents in pediatric oncology. These include the need for physicians to be honest about poor prognosis well [8]. Anticipatory information and guidance can empower family members. “Illness uncertainty” is a phrase intended to capture a person’s confusion about an illness. If a person finds an illness to be unpredictable, ambiguous, and/or incomprehensible, the person may experience illness uncertainty. Illness uncertainty has been studied and associated with parental adjustment outcomes [9]. Parent distress, post-traumatic stress symptoms, and parenting stress have been associated with illness uncertainty, as has the child’s general health-related quality of life. Clear, high-quality information and conversations shared between parent and provider may facilitate a parent’s realistic appraisal of a child’s health situation (also referred to as interpretive control or coping by gaining knowledge) and, consequently, decrease illness uncertainty. Parents, not being able to control their child’s health outcome, may employ interpretive control and positively impact their emotional adjustment [10]. Of note, honest prognostication, as well as eliciting and respecting the patient’s goals of care, has been associated with improved patient satisfaction with care [11]. Interestingly, it also fosters hope, even when prognosis is poor [12]. Hope takes on many forms. When cure cannot be attained, parents hope for more time, quality of life, and normalcy [13]. A clear message from parents was to support hope and realism as the two can stand side by side.

Conversely, inadequate preparation for death is apparently a common, recently reported experience among parents, noted by 40% of bereaved parents in a pediatric oncology setting [14]. The parents in this report, akin to those on our panel, felt specifically unprepared for the physical changes they witnessed as their child was dying. The dying trajectory or the decline toward death refers to the physical, spiritual, social, and psychological changes occurring proximal to death. Preparing for the imminence of death or diagnosing dying is an important component of anticipatory guidance and includes recognition of the specific signs of dying. By recognizing imminent death, care providers may optimize a patient’s comfort and dignity [15]. Touching and talking to the dying child may become a priority.

Parents on our panel expressed a desire for stepwise disclosure of information in order for them to prepare themselves to share the information with their children. This staged disclosure was also noted on interviews with parents of children with cancer on the topic of cancer diagnosis and treatment [16]. A parent’s role in supporting and guiding their child is facilitated by this carefully timed disclosure by professional caregivers [17]. Depending upon the child’s characteristics (e.g., maturation, experience with health condition, personality), parents may reinforce material information for the child and foster decision making outside of conversations with professional caregivers [18]. As effective communication is the cornerstone of family-centered care, previous interviews with bereaved parents of children with cancer revealed that parental ratings of care were directly related to their perceptions of the quality of communication among them, their children, and their physicians [19].

Browning and colleagues [3], who developed a communication training program entitled the Program to Enhance Relational and Communication Skills, proposed that medical professionals have been trained in part by a “hidden curriculum”. This implicit culture of medical training implies that “uncertainty and complexity are to be avoided”. Yet, from the perspective of parents, personalized care is preferred and includes complex, fluid, and evolving relationships. Emotional presence and authenticity, as well as confidence, creativity, and independent problem solving, may be needed in the provision of personalized care. Professional caregivers’ roles may shift during end-of-life care. Those with a chaplaincy training background may refer to one such important role as the “ministry of presence” [20]. Empathy and support toward patients and their families may become a more prominent function of the professional caregiver at the patient’s end of life, compared with early treatment, and may be among the professional’s most powerful tools [3]. Compassionate care begets an exclusive level of intimacy typically shared amongst family members and has been associated with parents’ psychological adjustment following their child’s cancer diagnosis [21].

This complex set of abilities needed in the end-of-life setting often requires collaboration of the interdisciplinary team, and, as these parents also noted, an interdisciplinary team is essential to palliative care. The formal training of each profession complements the other. For example, the psychosocial providers’ formal training focuses on communication skills and empathy; consequently, they may facilitate team, patient, and family dialogue and conferences [22]. The need for enhanced psychosocial support integrated into medical services is a recently identified theme among bereaved parents [14]. Since siblings of pediatric cancer patients are at risk for maladjustment, psychosocial services should also be provided to the siblings over the course of the illness and made available following death [23]. Another relevant issue raised by the parents was the need for alignment of the information provided by the interdisciplinary team. This is especially important when information is shared with children [24]. Both physicians and parents cite conflicting information as the most common miscommunication event that occurs in hospitalized children [25]. Not surprisingly, the receipt of conflicting information is associated with lower ratings of quality of care as reported by bereaved parents of children with cancer [19].

Parents also specifically discussed the importance of conversations about goals of care and advance directives in order to make plans. Goals of care are affected by health-related quality of life, more specifically one’s physical, psychological, social, and spiritual wellbeing, and incorporating these concepts into conversations about these goals may facilitate timely decision making [26]. Initiating end-of-life conversations as disease advances serves to prepare the parent and child. In fact, 75% of teens with cancer endorsed having advance care planning conversations throughout the cancer trajectory [27]. However, despite this clear endorsement from patients and caregivers, time spent in these discussions remains scant and late. A recent longitudinal study of conversations between oncologists and parents of children with advancing cancer revealed that <5% of time was spent discussing advanced directives and goals of care [28]. These conversations are difficult, and oncologists may feel unprepared/untrained to have them [29]. Our study narratives reveal that parents have insight to the difficulty that oncologists have in discussing end of life with them and that they (parents) might “yell and scream” at the messenger of bad news, but that they (parents) acknowledge the need to hear the message. They ask that providers not be afraid of them and remind providers that the emotions are not actually directed at the providers. The fear of giving bad news is pervasive in medicine and has many logical reasons; after all, bad news is bad [30]. Parents need us to be brave in the face of this bad news so that they can plan, prioritize, recalibrate their goals, and optimize the quality of life at end of life for their child. Sheryl Sandberg, in writing about her own development, described having coached herself to “lean in” upon facing a challenge, as opposed to shying away. Perhaps providers can aspire to broach the topic of death with less fear and even “lean in” toward death [31].

Consultation with pediatric palliative care providers also provides a means to meet this service gap where this resource is available. Indeed, earlier pediatric palliative care consultation is a predictor of earlier discussion of advance directives [32]. In addition, there exist guides for such conversations in adults, for example, the Serious Illness Conversation Guide [33], and efforts are currently underway to adapt this guide for use in the adolescent and young adult population [34].

Once parents and patients are given opportunities to discuss prognosis, goals of care, and an end-of-life plan, fostering compassionate care and comfort in the location of choice is crucial. Respecting autonomy is essential throughout the life span [35]. The dying patient has basic requirements that must be met, including “independence, dignity, acceptance by others of an individual approach to dying, relief of symptoms, and physical care” [36]. Hospitals may be the end-of-life location of choice for a variety of reasons, but they are not set up ideally to care for dying children [37]. Models of integration of primary palliative care and specialty palliative care into hospital settings have been proposed and piloted with success [38,39].

After the death of the child with cancer, meaningful contact between the healthcare team and bereaved parents, complemented with bereavement information and resources, positively impacts parents psychologically. Follow-up of bereaved parents is considered an evidence-based standard of care in the field [40]. The bereaved parent participants in this study also described the therapeutic role of serving on our Supportive Care Councils. Lastly, families want their children to be remembered. Memories have been likened to blessings. Remembering and actions of remembrance may facilitate the grief process [41].

Parents’ narratives as an education tool in palliative care may have a major advantage and complement more commonly available teaching modalities. Milota and colleagues (2019) conducted a systematic review on this subject and found a substantive body of work and positive training outcomes associated with narrative medicine [42]. Attitudes, knowledge, and skills were modified and enhanced shared decision making, meaningful relationships, ethical reasoning, and professional growth. Narrative-based teaching may be of particular benefit in palliative care training as its method cultivates skills that are particularly valuable in palliative care. That is to say, through this approach, psychological elements and medical knowledge are integrated to promote listening skills, shared understanding, empathy, and enhanced connection [4]

In conclusion, these bereaved parent narratives represent rich sources of information to guide oncologists and interdisciplinary healthcare providers by providing 10 essential communication and care themes pertaining to care before, during, and after the death of a child with cancer. Before death, parents emphasized honest, ongoing, and stepwise disclosure of prognostic information, support for hope and realism, anticipatory guidance for end-of-life planning, and support of an interdisciplinary team. During the dying process, they vocalized the need for support in ensuring their choice in location of death, and that comfort and compassion for the dying be prioritized in any location. After death, the bereaved want options to stay in contact with the healthcare team, to have opportunities to give back to the oncology community, and to ensure their child is remembered. Limitations of this report include the small parent sample and single institution. All parents had the experience of one comprehensive cancer center and had access to interdisciplinary psychosocial and palliative care teams. The information gleaned from these parents and others suggests that more research is needed on how to incorporate the experience and wisdom of bereaved parents into end-of-life communication training and skills. They should also inform studies that test the effects of educational interventions provided to clinicians on the care delivered to these patients and their parents.

## Figures and Tables

**Table 1:** Children represented.

Disease	Years of Age	Gender	Location of Death
neuroblastoma	16	f	home
leukemia	14	m	home
gliosarcoma	13	m	home
leukemia	11	f	home
ependymoma	7	f	hospital

**Table 2:** Temporally sequential themes: parent-identified essential end-of-life care elements.

Temporally Sequential Themes: Parent-Identified Essential End-of-Life Care Elements
Before Death
Invite open, ongoing communication and check for understanding; this is not a one-time conversation.
Enable stepwise disclosure; allow parents time to process news before telling the child, and allow the child to have a voice once told.
Ensure the availability and alignment of a multidisciplinary team.
Discuss all options for treatment, including second opinions; be willing to try new treatments.
Support hope, as well as realism; hope can be for different things (when cure is unrealistic).
Support conversations on goals of care and advance directives.
During Death
Provide anticipatory guidance on the process of dying, from physical changes to facilitating closure between the dying child and their siblings.
Comfort the dying patient regardless of location at EOL.
After Death
Support bereaved parents and provide opportunities for them to support other parents.
Honor their child’s legacy and keep their child’s memory alive.
